# 

**Published:** 1895-07-20

**Authors:** 


ABSOLUTELY PURE, therefore BEST.

**CADBURY'S COCOA**

"Of absolute purity and freedom from alkali."

—*Braithwaite.*

**CADBURY'S COCOA**

"The Typical Cocoa of English Manufacture Absolutely Pure."—*The Analyst.*

### THE HOSPITAL

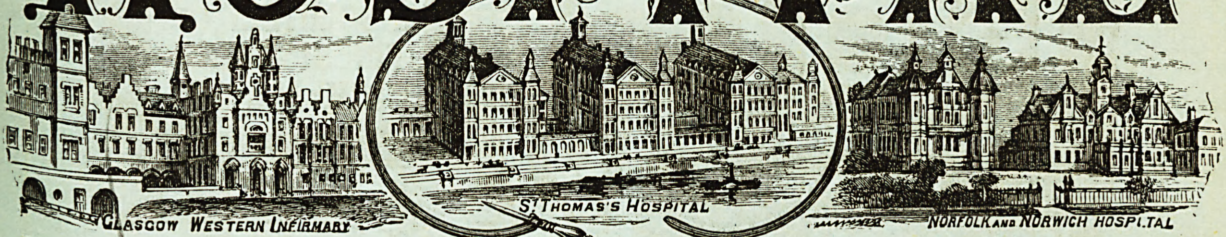

No. 460. Vol. XVIII.

Saturday, July 20th, 1895.

**WITH EXTRA NURSING SUPPLEMENT.**

[Registered at the General Post Office as a Newspaper for transmission in the United Kingdom and Abroad.]

